# Effects of norspermidine on *Pseudomonas aeruginosa* biofilm formation and eradication

**DOI:** 10.1002/mbo3.338

**Published:** 2016-01-27

**Authors:** Lin Qu, Pengfei She, Yangxia Wang, Fengxia Liu, Di Zhang, Lihua Chen, Zhen Luo, Huan Xu, Yong Qi, Yong Wu

**Affiliations:** ^1^ Department of Medicine Clinical LaboratoryThe Third Xiangya Hospital of Central South UniversityChangsha410013China; ^2^ Xiangya School of MedicineCentral South UniversityChangsha410013China

**Keywords:** Bacterial adhesion, biofilm, norspermidine, *Pseudomonas aeruginosa*, quorum sensing

## Abstract

Biofilms are defined as aggregation of single cell microorganisms and associated with over 80% of all the microbial infections. *Pseudomonas aeruginosa* is a Gram‐negative opportunistic pathogen capable of leading to various infections in immunocompromised people. Recent studies showed that norspermidine, a kind of polyamine, prevented and disrupted biofilm formation by some Gram‐negative bacterium. In this study, the effects of norspermidine on *P. aeruginosa* biofilm formation and eradication were tested. Microtiter plate combined with crystal violet staining was used to study the effects of norspermidine on *P. aeruginosa* initial attachment, then we employed SEM (scanning electron microscope), qRT‐PCR, and QS‐related virulence factor assays to investigate how norspermidine prevent biofilm formation by *P. aeruginosa*. We reported that high‐dose norspermidine had bactericide effect on *P. aeruginosa*, and norspermidine began to inhibit biofilm formation and eradicate 24‐h mature biofilm at concentration of 0.1 and 1 mmol/L, respectively, probably by preventing cell‐surface attachment, inhibiting swimming motility, and downregulating QS‐related genes expression. To investigate the potential utility of norspermidine in preventing device‐related infections, we found that catheters immersed with norspermidine were effective in eradicating mature biofilm. These results suggest that norspermidine could be a potent antibiofilm agent for formulating strategies against *P. aeruginosa* biofilm.

## Introduction

Biofilms are defined as aggregation of single cell microorganisms that are physiologically distinct from their free‐swimming counterparts. Biofilms are associated with over 80% of all the microbial infections associated with the urinary tract, catheters, dental plaque, and gingivitis (Girennavar et al. 2008). The major feature of biofilms is the presence of highly hydrated extracellular polymeric substance (EPS), including polysaccharides, proteins, and extracellular DNA (eDNA) (Billings et al. 2015). In terms of bacterial infections, biofilms can manifest as growth on medical devices or a range of host tissues. Compared to their planktonic counterparts, biofilm‐derived bacteria have distinctive phenotypes with regard to growth, gene expression, and protein production that enable the bacterial communities to persist and cause repeated waves of damage (Costerton et al. 1999; del Pozo and Patel 2007). Cells embedded in biofilms are up to 1000‐fold more resistant to antibiotics compared to their planktonic ones (Kouidhi et al. 2015). These complications lead to increased patient morbidity, increased costs of treatment, and higher rates of hospitalization (Costerton et al. 1999; Lister et al. 2009).

*Pseudomonas aeruginosa* is a Gram‐negative opportunistic pathogen commonly found in soil and water (Rahme et al. 1995), capable of leading to various infections in immunocompromised people, including bacteremia, sepsis, pneumonia, and wound and skin infections (Kipnis et al. 2006). It is a clinically important opportunistic pathogen responsible for 57% of total nosocomial infections (Sarabhai et al.^2013^). Besides the strong ability of *P. aeruginosa* to form biofilms in environments renders antibiotic treatments inefficient and lead to chronic infectious disease (Høiby et al. 2010; Bjarnsholt 2013). The pronounced ability of *P. aeruginosa* to develop biofilm‐associated disease has drawn considerable interest over the past decade in developing strategies to target their disassembly.

Since the increased resistance to antibiotics and conventional treatment of bacteria in biofilms, finding substances that can inhibit biofilm formation or trigger mature biofilm disassembly attracts considerable interest.

Norspermidine, a kind of polyamine, is a small organic hydrocarbon that is positively charged at physiological pH. Polyamines are pervasive at millimolar concentrations in both eukaryotes and prokaryotes and are required for normal cell growth (Tabor and Tabor 1984). Many research reported that norspermidine can enhance *Vibrio cholerae* biofilm formation (Parker et al. 2012; Karatan et al. 2005; McGinnis et al. 2009; Lee et al. ). However, Kolodkin‐Gal claimed that norspermidine was identified from the supernatants of disassembled biofilms and reported to mediate *Bacillus subtilis* (*B. subtilis*) biofilm disassembly by interacting with exopolysaccharide, but Kolodkin‐Gal's claims have been challenged by Hobley et al. (2014). Even so, recent publications have shown that norspermidine can prevent biofilms formation by a diverse range of bacterial species, including *Staphylococcus aureus* (Böttcher et al. 2013), *Staphylococcus epidermidis* (Ramón‐Peréz et al. 2015), *B. subtilis* (Böttcher et al. 2013), and *Escherichia coli* (Nesse et al. 2015).

Norspermidine is able to inhibit biofilm formation in some strains. However, to date there are no reports about the effect of norspermidine on *P. aeruginosa* clinical strains. The aim of the present study was to investigate whether the norspermidine may have potential as biofilm controlling substances against potentially pathogenic *P. aeruginosa* both in standard strain PAO1 and clinical isolates. The mechanism(s) of interactions were investigated via microtiter attachment assay, motility assays, and real‐time PCR (qPCR).

## Materials and Methods

### Bacterial strains and growth media

*Pseudomonas aeruginosa* strain PAO1 (ATCC 15692) is a well‐characterized wound isolate widely used as a laboratory strain (Holloway 1955; Schmidt et al. 1996). The clinical isolates utilized in this study were selected from a collection of clinical isolates of *P. aeruginosa* during January 2014 to December 2014 (She et al. 2015) from the Third Xangya hospital of Centre South University (Changsha, Hunan, China). No special ethical permit was required for this study according to the Chinese law. Pure stock cultures were maintained at −80°C in 30% (vol/vol) frozen glycerol solution. For planktonic growth, PAO1 and clinical strains of *P. aeruginosa* were cultured in Luria–Bertani broth (LB) at 37°C. Bacteria were subcultured on blood agar plates overnight at 37°C. LB broth was used for bacterial culture in all experiments unless otherwise specified. Norspermidine (Sigma‐Aldrich, St. Louis, MO) was used, and neutralized when necessary with NaOH (1 mol/L) (pH 7–7.4). The absorbance (A) or optical densities (OD) were measured by a spectrophotometer (Bio‐Tek, USA).

### Minimum inhibitory concentration (MIC) and minimum bactericidal concentration (MBC) assay

The MIC was determined for norspermidine by the broth dilution method (Cha et al. 2007) and was carried out in triplicate. The antibacterial activity was examined after incubation at 37°C for 16–18 h. MIC was determined as the lowest concentration of test samples that resulted in a complete inhibition of visible growth in the broth. MBC was determined on the basis of the lowest concentration of norspermidine that kills 99.9% of the test bacteria by plating out onto blood agar plate.

### Planktonic cell growth

The planktonic cell growth assay was modified from Huang and Li as described previously (Huang et al. 2014). Overnight‐grown PAO1 cells (OD630 was adjusted to 0.5) were diluted 1:100 in LB with 0, 4, 5, 6, and 7 mmol/L norspermidine. These samples were then grown in 50 mL centrifugal tubes (Corning/Costar, USA) at 37°C with agitation (160 rpm) and every hour aliquot of 150 *μ*L supernatant was carefully transferred to another microtiter plate. The planktonic culture turbidity was read at OD630 by the spectrophotometer.

To detect the CFU of PAO1 with norspermidine treatment, overnight‐grown PAO1 cells were diluted 1:100 in LB with 0, 4, 5, 6, and 8 mmol/L norspermidine. Triplicate samples were grown in the 50 mL centrifugal tubes at 37°C with agitation (160 rpm) and every hour aliquot of 100 *μ*L supernatant was diluted in saline (0.9% NaCl) at an appropriate dilution and spiral plated on blood agar plates by a glass rod. The plates were incubated for 24 h, and colonies were counted. The number of CFU/mL was calculated.

### Biofilm determination

To detect the inhibitory effect of norspermidine on biofilm formation of PAO1, the semiquantitative determination of biofilm formation was performed in microtiter plates as described earlier, with minor modifications (Nesse et al. 2015). About 4 *μ*L culture and 196 *μ*L LB with or without the norspermidine to be tested were added to each well in 96‐well cell culture plates (Corning/Costar, USA). After incubation at 37°C for 24 h, the plates were washed gently with 0.9% saline, dried for 30 min at 37°C, and stained with 200 *μ*L 0.5% (w/v) crystal violet solution. After staining, the plates were washed with the saline, 200 *μ*L 95% ethanol was added to dissolve stained dye, and OD_570 nm_ of the adhered and stained cells were measured using the spectrophotometer. Tests were performed in triplicate in at least three independent experiments.

For the mature biofilm disassembly valuation, primarily the mature biofilm was allowed to form as follow: a volume of 100 *μ*L of 1:200 diluted overnight bacterial culture was added to a 96‐well cell culture plate with LB and the plate was incubated at 37°C for 24 h. Later the wells were washed with 0.9% saline and norspermidine was incorporated into LB to different concentrations. The plate was incubated for another 24 h and biofilm formation was determined as described earlier.

### Biofilms on urinary catheter surfaces

There is currently no standardized method for the detection and analysis of biofilms on urinary catheter surfaces. The silicone Foley's catheter (Medtrue, Jiangsu, China) was carefully cut into 10‐mm long pipes, and disinfection was performed by soaking them into 75% ethanol for 30 min, followed by successive washes of the inner and outer surface with distilled water using a syringe with the tip of the needle at the upper end of the catheter. Once dried, the pipes can be used.

### Microtiter attachment assay

Experiment was conducted based on the method previously described by Santiago and Lim (Santiago et al. 2015a,b). A 96‐well microtiter plate (Corning/Costar, USA) was prepared with norspermidine at the following concentrations: 0, 2, 2.5, 3, 3.5, and 4 mmol/L. Aliquots of PAO1 suspension (adjust to 0.5 McFarland) were added to these wells. The plate was incubated for 2 h at 37°C. Following incubation, the wells were washed with saline and biomass of cell attachment was determined by crystal violet staining method as described earlier.

### Swimming motility assay

The motility assay was performed as described earlier, with minor modifications (Rashid and Kornberg 2000). Media used for assay was LB broth that contained 0.3% (wt/vol) agarose. Swimming plates were inoculated with bacteria from an overnight culture in blood agar plates at 37°C with a sterile toothpick. The plates were then incubated at 30°C for 14 h.

### Gene expression analysis

The qRT‐PCR assay was modified from Maisuria and Hosseinidoust as described previously (Maisuria et al. 2015). Bacterial cells were grown to an OD_630_ of 0.5–0.8 (10 h, 37°C, 150 rpm) in LB broth with or without subinhibitory (4 mmol/L) norspermidine. Total RNA was extracted using a E.Z.N.A. Total RNA Kit II (Omega Bio‐tek, Norcross, GA). RNA concentration was quantified by measuring the absorbance of the sample at 260 and 280 nm, and 1 *μ*L of RNA was used for cDNA synthesis using the TransScript All‐in‐One First‐Strand cDNA Synthesis SuperMix for qPCR (Transgene, Beijing, China). Expression of target genes was quantified using qRT‐PCR with the synthesized cDNA. qRT‐PCR was performed with a real‐time quantitative PCR system (Eppendorf, Germany) using TransStart^™^ Green qPCR SuperMix UDG (Transgene, Beijing, China). Conditions for qRT‐PCR were the following: 50°C for 2 min, initial denaturation at 94°C for 10 min, and 40 cycles of 5 sec at 94°C and 30sec at 60°C. Data were normalized to the endogenous reference gene of *P. aeruginosa*. The threshold cycle method (2^−ΔΔCT^) was used to analyze changes in gene expression in a given sample relative to the control. For each sample of cells, qRT‐PCR was performed in triplicate and the entire experiment was repeated twice with RNA samples extracted from independent cultures. The reported oligonucleotide primer sequences (Kim et al. 2015a,b) used to amplify the genes of interest are listed in Table 1.

**Table 1:** Primers used in this study

Primer name	Gene	Oligonucleotide sequences (5′→3′)	Tm (°C)	GC%	Product size (bp)
lasR‐F	*lasR*	ACGCTCAAGTGGAAAATTGG	60.11	45.00	247
lasR‐R	*lasR*	GTAGATGGACGGTTCCCAGA	59.93	55.00	
lasI‐F	*lasI*	CTACAGCCTGCAGAACGACA	60.20	55.00	168
lasI‐R	*lasI*	ATCTGGGTCTTGGCATTGAG	60.07	50.00	
rhlR‐F	*rhlR*	AGGAATGACGGAGGCTTTTT	60.07	45.00	231
rhlR‐R	*rhlR*	CCCGTAGTTCTGCATCTGGT	60.13	55.00	
rhlI‐F	*rhlI*	CTCTCTGAATCGCTGGAAGG	60.09	55.00	240
rhlI‐R	*rhlI*	GACGTCCTTGAGCAGGTAGG	59.87	60.00	
mvfR‐F	*mvfR*	AACCTGGAAATCGACCTGTG	59.97	50.00	238
mvfR‐R	*mvfR*	TGAAATCGTCGAGCAGTACG	60.01	50.00	
proC‐F	*proC* a	GGCGTATTTCTTCCTGCTGA	60.35	50.00	236
proC‐R	*proC* a	CCTGCTCCACTAGTGCTTCG	61.12	60.00	

a Housekeeping genes (endogenous control).

### Analysis of the production of QS‐related virulence factors

Overnight culture of *P. aeruginosa* was inoculated in LB broth with or without norspermidine (0, 2, 4 mmol/L) and incubated at 37°C for 16 h at 150 rpm. As described earlier (Sarabhai et al. 2013), pyocyanin pigment was extracted from culture supernatant by chloroform in the ratio of 3:2 and re‐extracted with 1.0 mL of 0.2 mol/L HCl and absorbance was read at 540 nm. For assaying elastase activity, 250 *μ*L elastin Congo red solution (5 mg/mL, pH 8) was incubated with 750 *μ*L cell‐free supernatant at 37°C for 16 h at 200 rpm. The mixture was centrifuged at 3000*g* for 10 min and absorbance was read at 490 nm. For assaying protease activity, azocasein solution (2%, pH 7) and culture supernatant were incubated at 37°C in 1:1 ratio for 1 h in a reaction volume of 400 *μ*L. The reaction was stopped by the addition of 500 *μ*L of 10% trichloroacetic acid and centrifuged at 8000*g* for 5 min to remove residual azocasein. The absorbance of supernatant was read at 400 nm.

### Effect of norspermidine on PAO1 biofilm morphology

PAO1 biofilms developed in the presence of norspermidine on glass covers for 12 h or 24 h were washed with saline and stained with crystal violet. After air drying, the glass covers were immediately processed for observation. Morphology of PAO1 in untreated and norspermidine‐treated biofilm was visualized and photographed with an Olympus CX31 light microscope (Tokyo, Japan). For mature biofilm disassembly valuation, 24‐h biofilms were treated with or without norspermidine for another 24 h, and were photographed with the microscope.

### Scanning electron microscope (SEM)

The bacterial biofilms colonizing the surfaces of the urinary catheters or cover glass were examined by SEM analysis. Sterile silicone Foley's catheters were gently cut into 1.0‐cm small pipes. For biofilm eradication assay, the pipes and cover slides were placed into six‐well cell culture plates with 2 mL LB broth containing 40 *μ*L overnight‐grown PAO1. After 24 h incubation, the culture media was removed and the pipes and cover slides were wash three times with 0.9% saline, then the pipes were transferred to a new six‐well plates containing 2 mL LB broth with or without norspermidine and incubated for another 24 h. Then, the pipes and cover slides were rinsed with saline and examined with SEM. Samples were fixed in a 2% glutaraldehyde and 0.1 mol/L cacodylate buffer (pH 7.4) for 30 min and then rinsed three times for 10 min each rinse in 0.2 mol/L cacodylate buffer (pH 7.4). Samples were then dehydrated by passing them through the following ethanol series: 30%, 50%, 70%, and 90% ethanol, each for 10 min, followed by 100% ethanol twice for 10 min each time. Support samples were then air dried in a desiccator for 24 h. After being coated with gold‐palladium, samples were examined under a S‐3400N scanning electron microscope (Hitachi, Japan).

### Statistical analysis

All experiments were performed in duplicate and repeated at least three times on different days. A two‐tailed Student's *t* test was used to determine whether the presence of norspermidine resulted in a significant difference compared to levels for the control. A *P *< 0.05 was considered significant. Data were analyzed using Graph Pad Prim 5.0 software (USA). All results are expressed as the mean ± standard deviation (SD).

## Results

### Norspermidine inhibits planktonic cell growth

**Figure 1:**
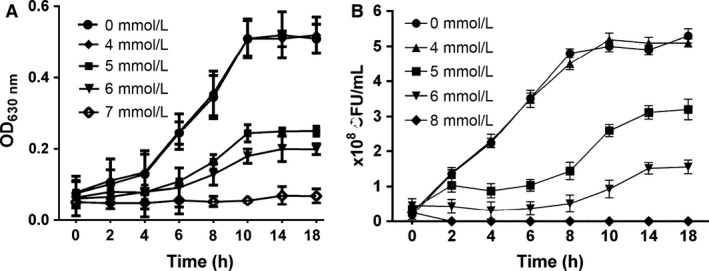
PAO1 planktonic cell growth with norspermidine. (A) Kinetic planktonic cell growth (optical density). Cells grown overnight were treated with 0, 4, 5, 6, and 7 mmol/L norspermidine, and every 2 h cells were carefully transferred to another microtiter plate for total 18 h, and OD_630 nm_ was read. (B) Planktonic cell growth (CFU). PAO1 cells grown overnight were treated with 0, 4, 5, 6, and 8 mmol/L norspermidine, and every 2 h cells were diluted and spiral plated on blood agar plates. Error bars represent SD.

The MIC and MBC of norspermidine were both 12.5 mmol/L. Norspermidine did not inhibit PAO1 kinetic planktonic cell growth at the sub‐MIC concentration that is equal or less than 4 mmol/L (Fig. 1A). At the same time, high dose of norspermidine (≥6 mmol/L) showed significant bactericide effect on planktonic cells, however, ≤4 mmol/L norspermidine did not show bactericide effect on the planktonic cells (Fig. 1B).

### Antibiofilm effect of norspermidine

**Figure 2:**
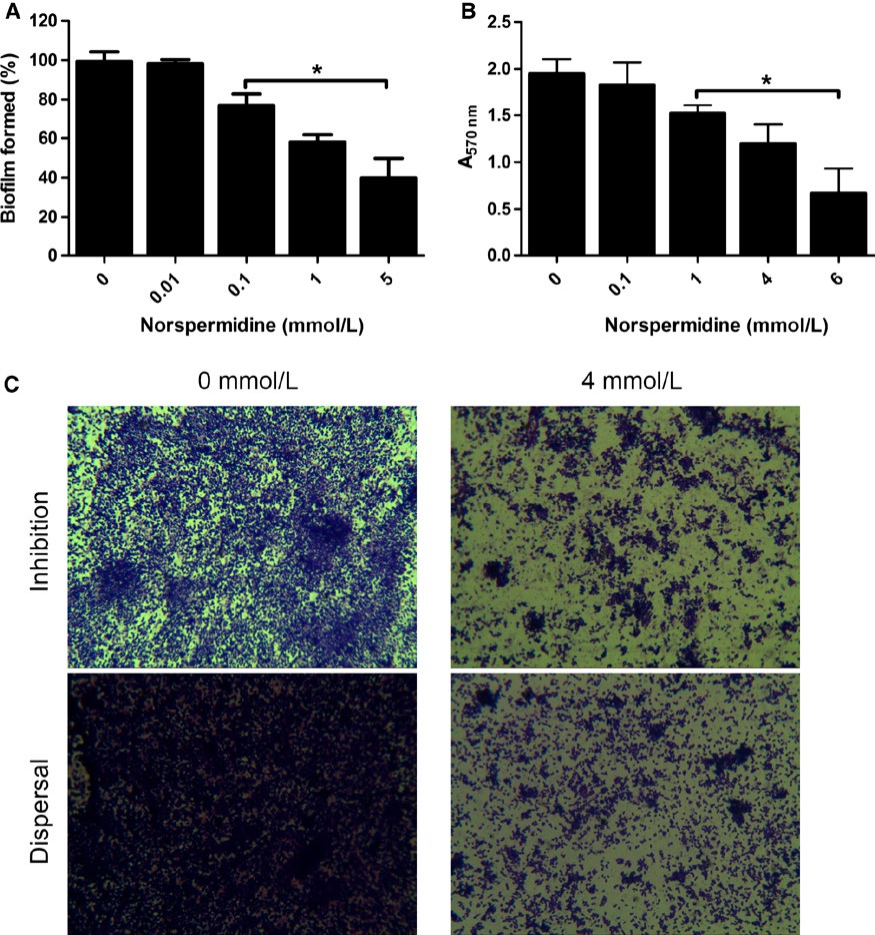
The effect of norspermidine against PAO1 biofilm. (A) Biofilm formation. Overnight culture was treated with 0–5 mmol/L norspermidine for 24 h. The volume of biofilm was determined by crystal violet assay. Error bars represent SD. (B) Biofilm eradication. Twenty‐four hour mature biofilms were cultured overnight with norspermidine at concentrations ranging from 0 to 6 mmol/L and the quantification of biofilm formation determined by crystal violet assay. *P < 0.05 versus untreated control. Error bars represent SD. (C) Representative images (magnification, 10 × 100) of PAO1 biofilm treated with norspermidine, and the effects of norspermidine were assessed by crystal violet staining.

To evaluate the potential clinical application of norspermidine, we tested whether norspermidine effectively inhibited biofilm formation or eradicated 24 h mature biofilms of standard strain PAO1 (Fig. 2A–C) and clinical isolates (Table 2). The effect of norspermidine on biofilms was evaluated by crystal violet assay. Biofilm biomass was checked by taking absorbance at 570 nm.

**Table 2:** Effects of norspermidine on clinical isolates of *Pseudomonas aeruginosa*

Clinical isolates	Biofilm (A_570 nm_)	% Inhibition^a^		% Disassembly^b^	
		5 mmol/L	10 mmol/L	10 mmol/L	25 mmol/L
PA01	1.058	2.84	**82.35**	**68.19**	**85.66**
PA02	1.050	**33.01**	**84.59**	**84.66**	**86.88**
PA04	1.174	1.01	**66.21**	**50.47**	**88.67**
PA08	0.319	2.12	**54.78**	**47.70**	**88.56**
PA10	0.999	**34.92**	**87.53**	**43.34**	**86.42**
PA16	1.006	**29.06**	**79.98**	0.60	**82.35**
PA29	1.333	**66.99**	**72.28**	**60.90**	**87.09**
PA30	1.507	**54.58**	**90.02**	**69.13**	**86.18**
PA32	1.505	**55.90**	**90.37**	**72.53**	**91.31**
PA38	1.495	**61.17**	**77.94**	**72.75**	**89.66**
PA40	1.138	**58.29**	**86.52**	**45.64**	**79.26**
PA41	1.900	**64.24**	**84.80**	**76.52**	**89.73**
PA47	2.372	**20.45**	**47.64**	**56.95**	**72.30**
PA49	0.488	**61.98**	**80.61**	**84.37**	**91.80**
PA52	1.966	**74.42**	**89.26**	**73.32**	**90.32**
PA58	0.331	**18.01**	**52.75**	**73.99**	**80.90**
PA60	1.629	**63.07**	**70.77**	**80.03**	**91.23**
PA62	1.806	**65.83**	**86.07**	**80.07**	**91.07**
PA68	1.861	**64.82**	**77.61**	**80.84**	**89.13**
PA71	1.878	**65.08**	**79.30**	**60.30**	**83.93**

The percentages in bold indicate significant difference (*P* < 0.05) between A_570 nm_ of treatment with norspermidine and of treatment without norspermidine. Biofilm inhibition and eradication by norspermidine was conducted as described in the Material and Methods section and the assays were performed in three independent assays.

a Percent inhibition was calculated with reference to measuring at A_570 nm_ of biofilm formed without norspermidine.

b Percent eradication was calculated with reference to measuring at A_570 nm_ of 24 h mature biofilm cultured without norspermidine.

Inhibitory activities of norspernidine on PAO1 were dose dependent. Norspermidine significantly decreased more than 20% biofilm formation of PAO1 at concentration more than 0.1 mmol/L. And it can effectively inhibit more than 50% biofilm formation compared with untreated group at concentration more than 5 mmol/L (Figs. 2A and C). Norspermidine eradicated biofilms also in a dose‐responsive manner. It began to dismantle the 24‐h mature biofilm at 1 mmol/L (Figs. 2B and C). When tested against the biofilms of clinical isolates of *P. aeruginosa*, norspermidine was effective at blocking formation of most biofilms as determined by the measurement of the biofilm biomass. In addition to blocking biofilm formation, norspermidine also significantly eradicated established biofilms by the clinical strains.

**Figure 3:**
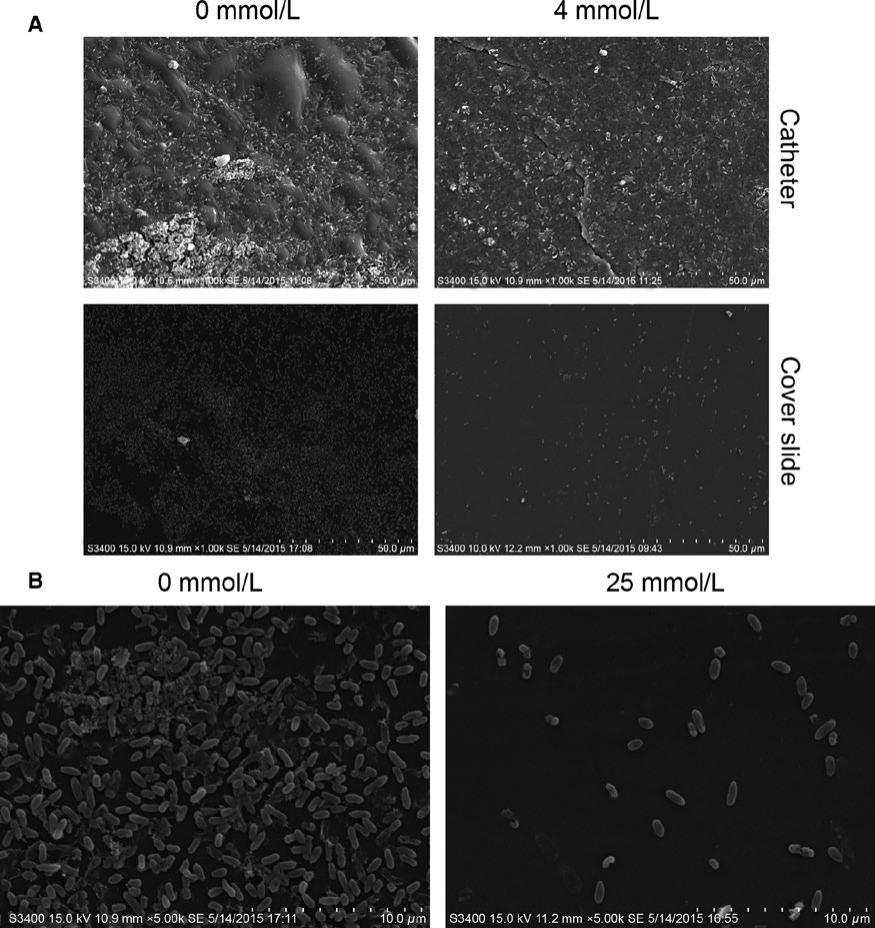
SEM examination for eradication of PAO1 mature biofilm by norspermidine. The 24 h mature biofilms on (A) catheter and cover slide were cultured with (4 mmol/L) or without norspermidine for another 24 h, then fixed and examined by SEM. (B) Norspermidine reduced the biofilm biomass and changed its morphology.

The strong antibiofilm activity was also observed by SEM. As shown in Figs. 3A, SEM was performed after norspermidine (4 mmol/L) treatment of 24 h old *P. aeruginosa* biofilms. Consistent with the observations acquired by crystal violet staining, SEM analysis also showed a dramatic reduction of biofilm attached to the surface of the urethral catheters and cover slides. Besides, biofilms treated with the norspermidine contained damaged bacteria (making the cells shrinking, diminished and ruptured) at high concentration (25 mmol/L), while these were not visible in nontreated biofilms (Fig. 3B), which suggest that high‐dose norspermidine would have a very similar bactericidal effect to that seen on planktonic cells.

### Effect of norspermidine on PAO1 attachment

**Figure 4:**
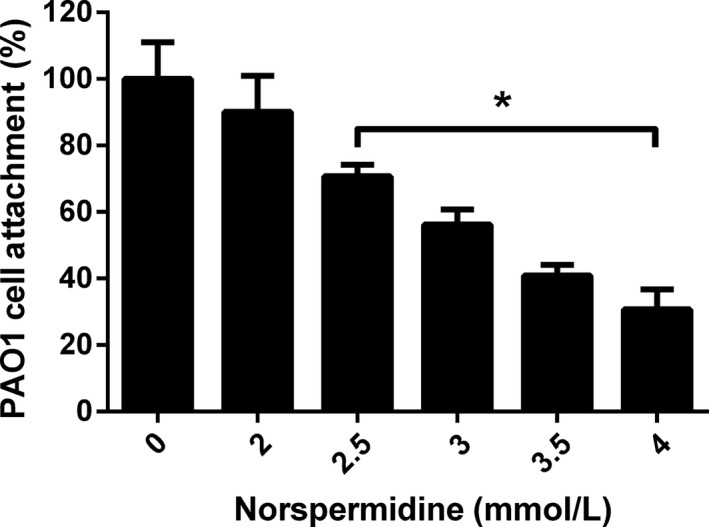
Attachment of PAO1 cells to the surfaces of microtiter plate wells containing norspermidine. Experiment is representative of three independent tests. *P < 0.05 versus untreated control, and error bars indicate SD.

We tested the inhibitory effect of norspermidine on the ability of *P*. *aeruginosa* to adhere to 96‐well plate. Cultures treated with norspermidine showed a concentration‐dependent reduction in cell‐surface attachment. Cell‐surface attachment reduction shows statistically significant (*P* < 0.05) at concentration of ≥2.5 mmol/L. Exposure to norspermidine at concentration of 4 mmol/L showed a 71.22% decrease in bacteria attachment as compared to control at 0 mmol/L (Fig. 4).

### Inhibitory effect of norspermidine on swimming motility

**Figure 5:**
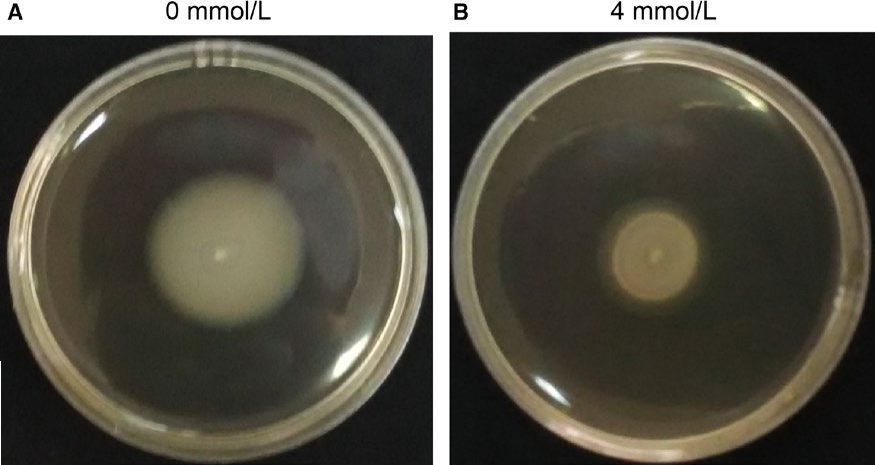
Photographs of plates showing inhibition of swimming motility of standard strain PAO1 in the presence of norspermidine (4 mmol/L).

Swimming motility zone of standard strain PAO1 without supplementation of norspermidine was 5.42 ± 0.51 cm that was significantly reduced to 3.12 ± 0.32 cm in the presence of 4 mmol/L norspermidine (Fig. 5). But norspermidine had few effects on swarming or twitching motilities (data not shown).

### Inhibitory effect of norspermidine on QS system

**Figure 6:**
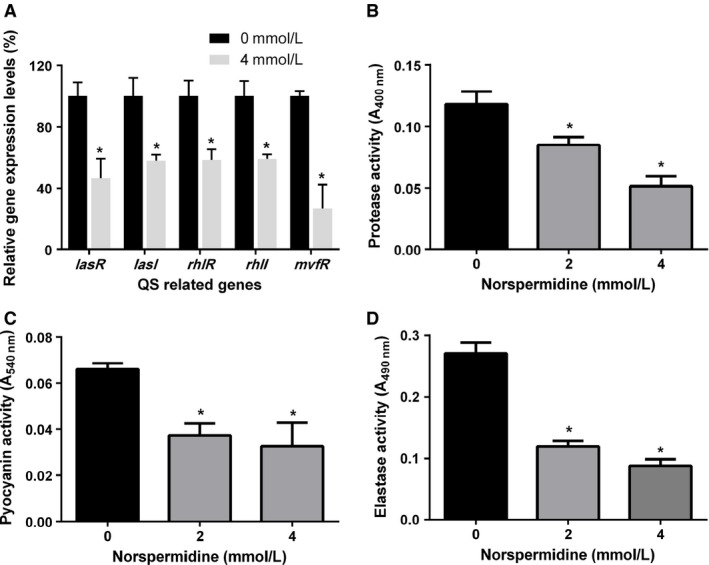
Norspermidne restrains the expression of QS‐related genes and production of virulence factors. (A) PAO1 was cultured and treated with 0 or 4 mmol/L norspermidine and qRT‐PCR analysis was performed as described in the Materials and Methods section. Activities of virulence factors including (B) protease activity, (C) pyocyanin, and (D) elastase activity at different concentrations of norspermidine (0, 2, and 4 mmol/L) were analyzed.*P < 0.05 versus untreated control, and error bars indicate SD.

To assess the effect of norspermidine on the gene expression of QS‐related genes, PAO1 was cultured in the presence of 4 mmol/L norspermidine and the gene expression was evaluated by real‐time PCR (Fig. 6A). The expression of *lasR*,* lasI*,* rhlR*,* rhlI*, and *mvfR*, which are related to QS system, was significantly decreased by norspermidine treatment at the concentration of 4 mmol/L. For better fold‐change comprehension, the traditional 2^−ΔΔCT^ value was calculated (Kim et al. 2015a,b). The gene expression analysis indicates that the norspermidine inhibit biofilm formation by downregulating the expression level of QS‐related genes.

At the same time, three QS‐related virulence factors (pyocyanin, elastase activity, and protease activity) were analyzed to assess the effects of norspermidine on virulence. Production of the three virulence factors was reduced by norspermidine in a concentration‐dependent manner (0, 2, and 4 mmol/L norspermidine added): 45–54% suppression for pyocyanin, 59–69% suppression for elastase activity, and 53–66% suppression for protease activity (Fig. 6B–D).

## Discussion

In this work, we demonstrated the antimicrobial and antibiofilm effects of exogenous norspermidine against *P. aeruginosa*. The effects of norspermidine on our *P. aeruginosa* strains was similar to that reported by *V. cholerae*,* Neisseria gonorrhoeae*,* B. subtilis*,* E. coli*,* S. aureus*,* S. epidermidis*, and even mixed culture (McGinnis et al. 2009; Böttcher et al. 2013; Ramón‐Peréz et al. 2015; Goytia et al. 2013; Si et al. 2015), although higher concentrations of norspermidine were required in our experiment. Furthermore, to our knowledge, there are no studies demonstrating the disassembly on biofilms of PAO1 and *P. aeruginosa* clinical isolates by norspermidine and its potential effect on bacterial adherence and QS‐related genes (*rhl*I/R, *las*I/R, and *mvf*R) expression and QS‐related virulence factors (pyocyanin, elastase activity, and protease activity). Therefore, this is the first work that highlights this effect.

Norspermidine could interact directly with the extracellular polysaccharide through binding to the functional group C‐O‐C. Furthermore, norspermidine could increase cell surface‐negative charge, decrease cell hydrophobicity (Si et al. 2014), and these features could contribute to the disassembly of biofilms.

In addition, biofilm formation by *P. aeruginosa* was also inhibited by treatment with norspermidine cultured both on polystyrene slide covers and on surface of catheter pieces (Fig. 3A). Many studies reported that norspermidine did not inhibit cell growth even at millimolar concentrations (Rasamiravaka et al. 2015), but our results showed that growth of *P. aeruginosa* was suppressed by treatment with high concentration of norspermidine (Fig. 1A). The bacterial counting assay also showed that norspermidine has an antibactericidal effect against *P. aeruginosa* (Fig. 1B). This may be due to differences between the strains tested. The variation in levels of clinical isolates sensitivity to norspermidine could be explained by the different biochemical composition in biofilms or different content of exopolysaccharides and the different structural arrangement of exopolysaccharide within the biofilm (Ramón‐Peréz et al. 2015). Furthermore, some strains may produce a more robust biofilm, making them more resistant to norspermidine.

There are essentially two major steps in biofilm production: (1) cell‐surface attachment, biofilm formation starts with adhesion of so‐called “linking film” bacteria, which provide the groundwork for further biofilm growth; (2) cell–cell interaction, which is an accumulative phase where the bacteria form microcolonies for construction of multilayer structure leading to biofilm development (Mack 1999; Götz 2002; Mack et al. 2004). As demonstrated by the biofilm microtiter attachment assay, biofilm inhibitors like norspermidine facilitate the separation of biofilms from surfaces. The percentage of bacterial cell‐surface attachment is directly proportional to the final mass of biofilm formed. A lower percentage of cell‐surface attachment therefore corresponds to a lesser number of bacterial cells involved in biofilm development, which essentially gives rise to formation of weaker biofilm structures, and making the bacteria more vulnerable to eradication (Santiago et al. 2015a,b). This property can be applied industrially, for example, to the removal of biofilm formed on membrane filters for water treatment (Kappachery et al. 2010) or on water/oil pipes.

Biofilm formation is invariably preceded by attachment, which is mediated by flagellar motilities (swimming and swarming) and in later stages by twitching motility (Klausen et al. 2003). Twitching motility occurs by successive extension and retraction of polar type IV pili but not flagella (Mattick 2002). Twitching motility appears to be predominantly a mean of rapid colonization by bacterial communities to new surface, and twitching motility is necessary for the assembly of a monolayer of *P. aeruginosa* cells into microcolonies (O'Toole and Kolter 1998; O'Toole et al. 1999). The swarming motility is a form of organized surface translocation which depends on extensive flagellation and cell‐to‐cell contact, and regulated by the *rhl* system (Daniels et al. 2004). Swarming motility is implicated in early stages of *P. aeruginosa* biofilm establishment. Strains grown under the conditions that promote swarming motility form flat and uniform biofilm while strains with limited swarming motility result in biofilm containing discontinuous cell aggregates (Rasamiravaka et al. 2015; Shrout et al. 2006). Although all those motilities are important for *P. aeruginosa* biofilm establishment, but our studies showed that norspermidine could nearly only inhibit swimming motility of *P. aeruginosa*.

Evidence is accumulating showing that the ability to form biofilms in many organisms involves QS regulation. *Pseudomonas aeruginosa* produces both cell‐associated and extracellular virulence factors (e.g., *lasB*‐elastase, *lasA*‐staphylolytic protease, *toxA*‐exotoxin A, and *aprA*‐alkaline protease) (Hentzer et al. 2002) globally regulated by QS systems arranged in hierarchical manner with *las* system at the top, positively controlling the activity of *rhl* system. QS circuits allow bacteria to coordinate their gene expression in a cell density‐dependent manner. The *las* system utilizes *N*‐(3‐oxododecanoyl)‐l‐homoserine lactone (3‐oxo‐C_12_HSL), whereas *rhl* system functions by means of *N*‐butanoyl‐l‐homoserine lactone (C_4_HSL) as the signal molecules (Rasamiravaka et al. 2015). In this study, to evaluate correlation between inhibitory effect by norspermidine and QS system, we determined the mRNA expression level of several representative QS‐related genes such as *lasR/I*,* rhlR/I*, and *mvfR*, using a real‐time PCR analysis. It is known that the *las* system (LasR/3‐oxo‐C_12_‐HSL) is essential for biofilm differentiation and plays a role during the irreversible attachment stage, whereas the *rhl* system (RhlR/C_4_‐HSL) is important for optimal biofilm formation (Favre‐Bonté et al. 2003). In this study, norspermidine significantly inhibited the transcription level of *lasR/I*,* rhlR/I*, and *mvfR*, and which appears to affect characteristic phenotypes as demonstrated by reduced biofilm formation, decreased bacterial motilities, and attachment.

At the same time, new important discoveries continue to be made, such as the recent determination that a library of compound that structurally mimicked norspermidine (like guanidine and biguanide) was synthesized chemically, which inhibited biofilm formation and disrupted existing biofilms by *B. subtilis* and *S. aureus* through binding to negatively charged or possibly polar groups through coulombic attraction and hydrogen bonding (Böttcher et al. 2013). Another example is that *B. subtilis* produces d‐amino acids in stationary phase and that they have biofilm inhibitory properties (Si et al. 2014; Hochbaum et al. 2011; Sanchez et al. 2013; Kolodkin‐Gal et al. 2010) without influence the growth of bacteria. Mixtures of d‐amino acids with norspermidine were found to be highly synergistic in disrupting biofilms (Si et al. 2014).

We conclude that norspermidine could inhibit biofilm formation and eradicate 24‐h mature biofilm in *P. aeruginosa* PAO1 and clinical strains by (1) preventing cell‐surface attachment; (2) inhibiting swimming motility phenotype; and (3) downregulating QS‐related gene expression. These results suggest that norspermidine could be a potent antibiofilm agent for formulating strategies against *P. aeruginosa* biofilm.

## Conflict of Interest

None declared.
